# Page Kidney Resulting From Traumatic Subcapsular Renal Hematoma: A Case Report

**DOI:** 10.7759/cureus.102236

**Published:** 2026-01-24

**Authors:** Anas E Ahmed, Rima A Asiri, Mashael A Alturki, Rawan M Alatawi, Abdelazeez I Alhisar

**Affiliations:** 1 Community Medicine, Jazan University, Jazan, SAU; 2 College of Medicine, King Khalid University, Abha, SAU; 3 College of Medicine, King Faisal University, Al-Ahsa, SAU; 4 Faculty of Medicine, University of Tabuk, Tabuk, SAU; 5 Faculty of Medicine, King Faisal University, Al-Ahsa, SAU

**Keywords:** acute flank pain, conservative management, minor blunt trauma, page kidney, renal compression, renal imaging, renin–angiotensin–aldosterone system, secondary hypertension, subcapsular renal hematoma

## Abstract

Page kidney is an uncommon but clinically important cause of secondary hypertension resulting from external compression of the renal parenchyma, most often due to a subcapsular or perinephric hematoma, leading to reduced renal perfusion and activation of the renin-angiotensin-aldosterone system. We report a case of a 45-year-old Middle Eastern male who presented with acute left flank pain and newly diagnosed severe hypertension following minor blunt trauma. Initial evaluation revealed localized flank tenderness and elevated blood pressure in the absence of hemodynamic instability or significant laboratory abnormalities. Imaging with ultrasonography demonstrated a compressive perinephric collection, while contrast-enhanced computed tomography confirmed a left-sided subcapsular renal hematoma causing significant parenchymal compression without evidence of active bleeding or vascular injury. Based on the clinical and radiological findings, a diagnosis of Page kidney was established. The patient was successfully managed conservatively with close inpatient monitoring, analgesia, and renin-angiotensin system-targeted antihypertensive therapy, resulting in progressive symptom resolution and blood pressure control. Follow-up imaging demonstrated partial hematoma resolution with preserved renal function. This case emphasizes that Page kidney can occur even after low-impact trauma and may present with subtle clinical findings aside from acute hypertension. Early recognition through careful blood pressure assessment and appropriate imaging is critical, as timely conservative management can lead to favorable outcomes and prevent long-term renal damage or persistent hypertension.

## Introduction

Page kidney is a rare but clinically significant cause of secondary hypertension that results from external compression of the renal parenchyma, leading to reduced renal perfusion and activation of the renin-angiotensin-aldosterone system (RAAS) [1,2]. The condition was first described by Irvine H. Page in 1939 following experimental compression of the kidney, which demonstrated a direct relationship between renal ischemia and hypertension [1-5]. Page kidney most commonly occurs due to subcapsular or perinephric hematoma, although other compressive etiologies such as urinomas, lymphoceles, tumors, or postoperative collections have also been reported [1,3]. While traditionally associated with high-energy trauma, renal biopsy, or surgical interventions, Page kidney can also arise after seemingly trivial or minor trauma, making early recognition challenging [2,4].

The clinical presentation of Page kidney is often variable and nonspecific, ranging from flank pain and hematuria to isolated new-onset or refractory hypertension [1,3]. Delayed diagnosis may result in persistent hypertension and irreversible renal damage if prolonged parenchymal compression occurs [1-3]. Imaging plays a pivotal role in diagnosis, with ultrasonography serving as an initial screening modality and contrast-enhanced computed tomography providing definitive characterization of the underlying cause and extent of renal compression [4,5]. Management strategies are guided by the severity of hypertension, renal function impairment, and the presence of ongoing bleeding, and may range from conservative medical therapy to interventional drainage or surgical decompression [6-8]. This case highlights the importance of maintaining a high index of suspicion for Page kidney even after minor blunt trauma, particularly in patients presenting with acute flank pain and unexplained hypertension.

## Case presentation

A 45-year-old Middle Eastern male patient presented to the emergency department with an acute onset of left flank pain following a minor blunt trauma. The patient reported that the injury occurred after a low-impact mechanical fall at home, during which the left flank struck the edge of a table. There was no history of high-energy trauma, loss of consciousness, or penetrating injury. The pain began shortly after the incident, progressively increased in intensity, and was described as constant, deep, and non-radiating. It was associated with nausea but no vomiting. The patient denied hematuria, dysuria, urinary frequency, fever, chills, chest pain, shortness of breath, or neurological symptoms. There was no prior history of hypertension, renal disease, bleeding disorders, anticoagulant use, or recent invasive procedures. Family history was unremarkable for renal or cardiovascular disease. The patient did not smoke, consume alcohol excessively, or use illicit drugs.

On initial examination, the patient was alert and oriented, appearing uncomfortable due to pain. Vital signs revealed elevated blood pressure measured at 178/102 mmHg, which was previously undocumented, a heart rate of 96 beats per minute, a respiratory rate of 18 breaths per minute, oxygen saturation of 99% on room air, and an oral temperature of 36.8°C. Cardiovascular and respiratory examinations were unremarkable. Abdominal examination revealed localized tenderness over the left flank and left upper quadrant without guarding or rebound tenderness. There was no palpable abdominal mass, organomegaly, or evidence of peritonitis. Costovertebral angle tenderness was present on the left side. Examination of the skin showed no ecchymosis or abrasions. Peripheral pulses were intact, and no peripheral edema was noted. Neurological examination was normal.

Initial laboratory investigations demonstrated a hemoglobin level of 12.8 g/dL, which was stable compared with baseline, and a normal platelet count. White blood cell count was mildly elevated at 11.2 ×10⁹/L. Serum creatinine was 1.1 mg/dL with an estimated glomerular filtration rate within normal limits. Blood urea nitrogen, electrolytes, liver function tests, and coagulation profile were within normal ranges (Table 1). Urinalysis revealed no gross hematuria, with only trace red blood cells on microscopy. Serum lactate was normal, suggesting hemodynamic stability.

**Table 1:** Summary of laboratory investigations obtained at initial presentation. Laboratory values demonstrate preserved renal function, stable hemoglobin levels without evidence of significant hemorrhage, normal coagulation parameters, and absence of clinically significant electrolyte or hepatic abnormalities. Mild leukocytosis was noted, likely reflecting a stress response. These findings supported hemodynamic stability and guided conservative management. Abbreviations: HPF, high-power field.

Laboratory test	Result	Reference range
Hemoglobin	12.8	12.0–16.0 g/dL
Hematocrit	38.5	36–46%
Red blood cell count	4.4	4.0–5.2 ×10⁶/µL
Mean corpuscular volume	87	80–96 fL
Mean corpuscular hemoglobin	29	27–33 pg
Mean corpuscular hemoglobin concentration	33	32–36 g/dL
Red cell distribution width	13.2	11.5–14.5%
White blood cell count	11.2	4.0–10.0 ×10⁹/L
Neutrophils	72	40–75%
Lymphocytes	20	20–45%
Monocytes	6	2–10%
Eosinophils	2	1–6%
Basophils	<1	0–1%
Platelet count	265	150–400 ×10⁹/L
Serum creatinine	1.1	0.6–1.3 mg/dL
Blood urea nitrogen	14	7–20 mg/dL
Sodium	139	135–145 mmol/L
Potassium	4.2	3.5–5.1 mmol/L
Chloride	102	98–107 mmol/L
Bicarbonate	24	22–29 mmol/L
Calcium	9.3	8.6–10.2 mg/dL
Aspartate aminotransferase	22	10–40 U/L
Alanine aminotransferase	19	7–56 U/L
Alkaline phosphatase	84	44–147 U/L
Total bilirubin	0.7	0.2–1.2 mg/dL
Albumin	4.1	3.5–5.0 g/dL
Prothrombin time	12.1	11–13.5 sec
International normalized ratio	1	0.9–1.1
Activated partial thromboplastin time	30	25–35 sec
C-reactive protein	4	<5 mg/L
Erythrocyte sedimentation rate	12	0–20 mm/hr
Serum lactate	1.1	0.5–2.2 mmol/L

**Figure 1:**
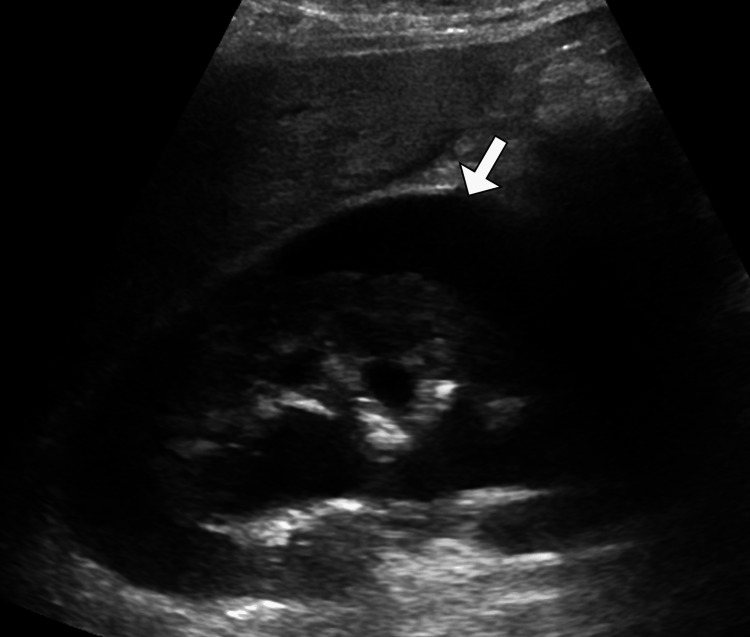
Left perirenal collection on greyscale ultrasound. Greyscale ultrasound image of the left kidney demonstrates a well-defined hypoechoic perirenal collection (arrow) surrounding the renal capsule. The appearance is consistent with a perirenal fluid collection in the clinical context of idiopathic intracranial hypertension.

**Figure 2:**
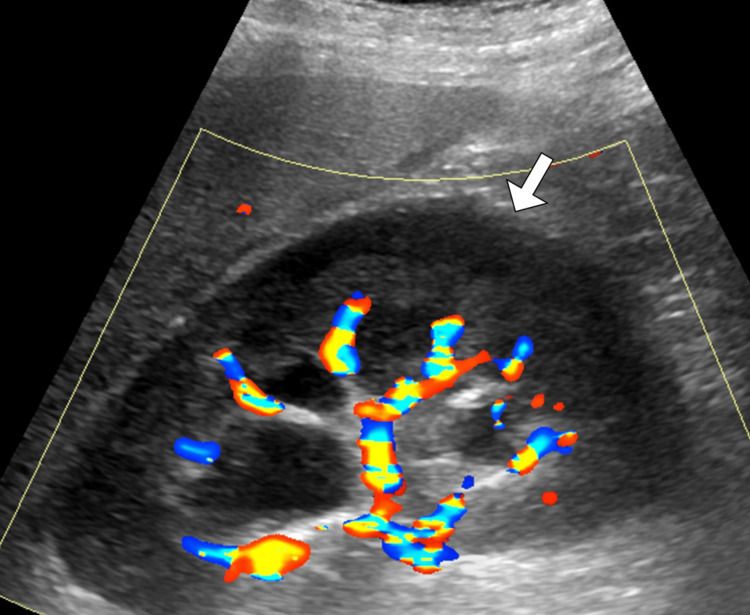
Color Doppler ultrasound of left perirenal collection. Color Doppler ultrasound image of the left kidney shows the previously identified perirenal collection (arrow) without internal vascularity. The absence of Doppler flow helps exclude a vascular lesion, supporting the diagnosis of a perirenal hematoma.

**Figure 3:**
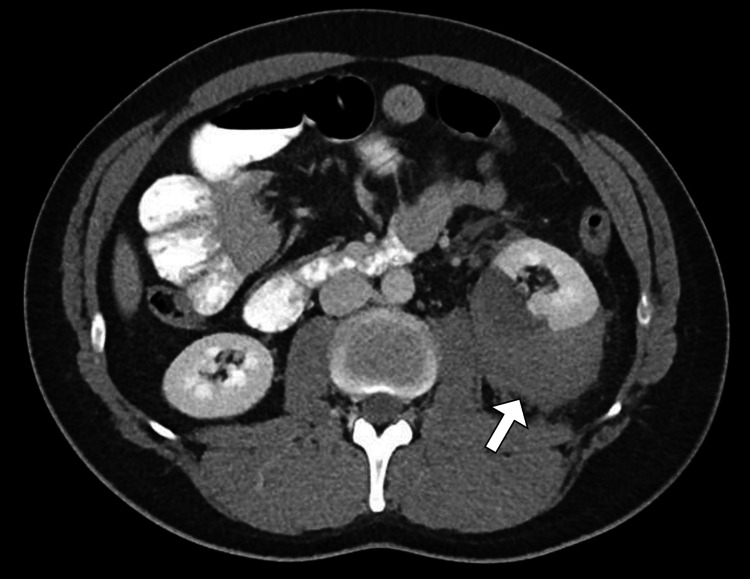
Left perirenal hematoma on axial CT. Axial contrast-enhanced CT image of the abdomen demonstrates a hyperdense left perirenal collection (arrow), consistent with a perirenal hematoma. This finding correlates with the ultrasound appearances and supports the diagnosis in the setting of idiopathic intracranial hypertension.

Given the combination of flank pain and newly detected hypertension following trauma, imaging was pursued. Renal ultrasonography demonstrated an enlarged left kidney with a hypoechoic, crescent-shaped perinephric collection causing compression of the renal parenchyma (Figure 1). Doppler assessment showed reduced intrarenal arterial flow with elevated resistive indices on the affected side, while the right kidney appeared normal (Figure 2). To further characterize these findings, contrast-enhanced computed tomography of the abdomen and pelvis was performed. CT imaging revealed a substantial left-sided subcapsular renal hematoma exerting mass effect on the renal parenchyma, with flattening of the kidney contour (Figure 3). There was no evidence of active contrast extravasation, renal laceration extending into the collecting system, vascular injury, or underlying renal mass. The renal artery and vein were patent. These imaging findings, in conjunction with acute hypertension, were highly suggestive of Page kidney.

The differential diagnosis initially included renal contusion, renal laceration, perinephric abscess, spontaneous retroperitoneal hemorrhage, renal artery thrombosis, and acute pyelonephritis. However, the absence of fever, normal inflammatory markers, lack of infectious features, preserved renal perfusion in major vessels, and the characteristic subcapsular hematoma with secondary hypertension helped exclude these conditions. The constellation of recent trauma, compressive perirenal hematoma, and new-onset hypertension led to the definitive diagnosis of Page kidney involving the left kidney.

Management was guided by the patient’s hemodynamic stability, preserved renal function, and absence of ongoing bleeding. A conservative approach was adopted. The patient was admitted for close monitoring, including serial blood pressure measurements, renal function tests, and hemoglobin levels. Antihypertensive therapy was initiated using an angiotensin-converting enzyme inhibitor to counteract RAAS activation. Adequate analgesia was provided, and physical activity was restricted. Surgical or interventional radiology consultation was obtained, and it was agreed that invasive intervention was not immediately indicated due to the lack of active bleeding or renal compromise.

During the hospital course, the patient’s pain gradually improved over several days, and blood pressure showed progressive control with medical therapy. Serial laboratory tests demonstrated stable hemoglobin and renal function. Repeat ultrasonography before discharge showed no increase in the size of the hematoma and mildly improved intrarenal blood flow. The patient was discharged in stable condition with antihypertensive medication, analgesics as needed, and clear instructions for outpatient follow-up. At follow-up four weeks later, the patient reported significant symptom resolution. Blood pressure was well controlled on a single agent, and renal function remained normal. The patient continued to be monitored to ensure complete hematoma resolution and reassessment of long-term blood pressure control.

## Discussion

Page kidney represents an uncommon but important etiology of secondary hypertension, arising from sustained external compression of the renal parenchyma that culminates in renal hypoperfusion and activation of the RAAS [3,6,8]. Although classically described following major renal trauma or iatrogenic interventions such as renal biopsy or surgery, increasing evidence suggests that even minor blunt trauma can precipitate this condition, particularly when subcapsular hematoma formation occurs [2-7]. The present case underscores the clinical relevance of Page kidney in the setting of low-impact injury and highlights the diagnostic and therapeutic challenges associated with its often subtle and nonspecific presentation.

The pathophysiology of Page kidney is well established and centers on mechanical compression of the kidney, which leads to reduced renal perfusion pressure and subsequent ischemia [3,6]. This ischemic insult stimulates excess renin release from the juxtaglomerular apparatus, resulting in systemic vasoconstriction, sodium and water retention, and ultimately hypertension [3,5]. If unrecognized or untreated, prolonged compression may lead to irreversible parenchymal damage, chronic kidney disease, or persistent hypertension even after resolution of the inciting lesion [4,5]. Notably, the degree of hypertension does not always correlate with the size of the hematoma, emphasizing that even modest collections can have significant physiological consequences, particularly when confined beneath the renal capsule [3-7].

Clinically, Page kidney may present with flank pain, abdominal discomfort, hematuria, or, as in some cases, isolated new-onset or refractory hypertension [6,7]. The absence of hematuria or overt signs of renal injury, especially after minor trauma, can delay diagnosis [1,5,8]. This highlights the importance of maintaining a high index of suspicion in patients presenting with unexplained hypertension in conjunction with flank pain or a recent history of trauma [3-7]. In the current case, the detection of acute hypertension in a previously normotensive patient was a critical clue that prompted further imaging and led to a timely diagnosis.

Imaging plays a central role in confirming Page kidney and differentiating it from other causes of secondary hypertension or post-traumatic renal pathology [1-7]. Ultrasonography is a valuable initial modality due to its accessibility and ability to identify perirenal collections, renal enlargement, and altered intrarenal Doppler flow patterns [3,4,6]. However, contrast-enhanced computed tomography remains the gold standard for diagnosis, as it provides superior delineation of subcapsular versus perinephric hematomas, assesses renal parenchymal integrity, excludes active bleeding or vascular injury, and identifies potential underlying lesions such as tumors or cysts [5-8]. In this case, CT imaging was instrumental in confirming the diagnosis and guiding conservative management by demonstrating the absence of active extravasation or major renal disruption.

The differential diagnosis of acute flank pain and hypertension following trauma is broad and includes renal contusion or laceration, renal artery thrombosis or dissection, spontaneous retroperitoneal hemorrhage, acute pyelonephritis, and adrenal pathology [1-4]. Page kidney should be distinguished from these entities, as management strategies differ substantially [3-10]. The characteristic finding of a compressive subcapsular hematoma with preserved renal vasculature strongly supports the diagnosis and helps avoid unnecessary invasive interventions.

Management of Page kidney is individualized and depends on hemodynamic stability, renal function, severity of hypertension, and the evolution of the compressive lesion [1,5,7]. Conservative treatment with antihypertensive therapy targeting the RAAS is appropriate in stable patients without evidence of ongoing bleeding or progressive renal impairment [4,6]. Angiotensin-converting enzyme inhibitors or angiotensin receptor blockers are particularly effective, as they address the underlying pathophysiological mechanism [1,4]. Invasive options, including percutaneous drainage, surgical capsulotomy, or nephrectomy, are reserved for cases with refractory hypertension, worsening renal function, infected collections, or failure of conservative therapy [2-7]. The favorable outcome in the present case supports a conservative approach in carefully selected patients, even in the presence of a significant hematoma, provided close monitoring is ensured.

## Conclusions

Page kidney should be recognized as a rare but important and potentially reversible cause of secondary hypertension, even following minor blunt trauma. This case highlights that subtle renal injury with subcapsular hematoma formation can result in significant renal compression and acute hypertension in otherwise stable patients. A high index of suspicion, careful blood pressure assessment, and timely imaging are essential for early diagnosis. Conservative management with close monitoring and targeted antihypertensive therapy can be effective in selected cases, preventing unnecessary invasive intervention and reducing the risk of long-term renal damage or persistent hypertension. Early recognition and individualized management remain the cornerstone for optimal outcomes in patients with Page kidney.
